# P-2004. Performance of a Metagenomic Next-Generation Sequencing Assay for Cerebrospinal Fluid Compared to Conventional Testing in Pediatric Patients

**DOI:** 10.1093/ofid/ofaf695.2168

**Published:** 2026-01-11

**Authors:** Samuel Showalter, Kevin Messacar, Elaine Dowell, Timothy M Blicharz, Steve Miller, Sudeb Dalai, Samuel R Dominguez

**Affiliations:** Colorado Children's Hospital, Aurora, CO; University of Colorado, Children’s Hospital Colorado, Aurora, Colorado; Children's Hospital Colorado, Aurora, Colorado; Delve Bio, Boston, Massachusetts; Delve Bio, Boston, Massachusetts; Delve Bio, Boston, Massachusetts; University of Colorado School of Medicine, Aurora, CO

## Abstract

**Background:**

Metagenomic next-generation sequencing (mNGS) enables unbiased detection of RNA and DNA viruses, bacteria, fungi, and parasites from cerebrospinal fluid (CSF). An mNGS assay developed at the University of California, San Francisco (UCSF) demonstrated potential clinical utility for suspected CNS infections. Delve Bio Inc. (Delve), under exclusive license from UCSF, has further refined the assay into a clinical commercial platform with a reduced turnaround time of approximately 2 days. This study evaluates performance of the updated mNGS platform using archived CSF samples from pediatric patients with suspected CNS infections.

**Figure:**
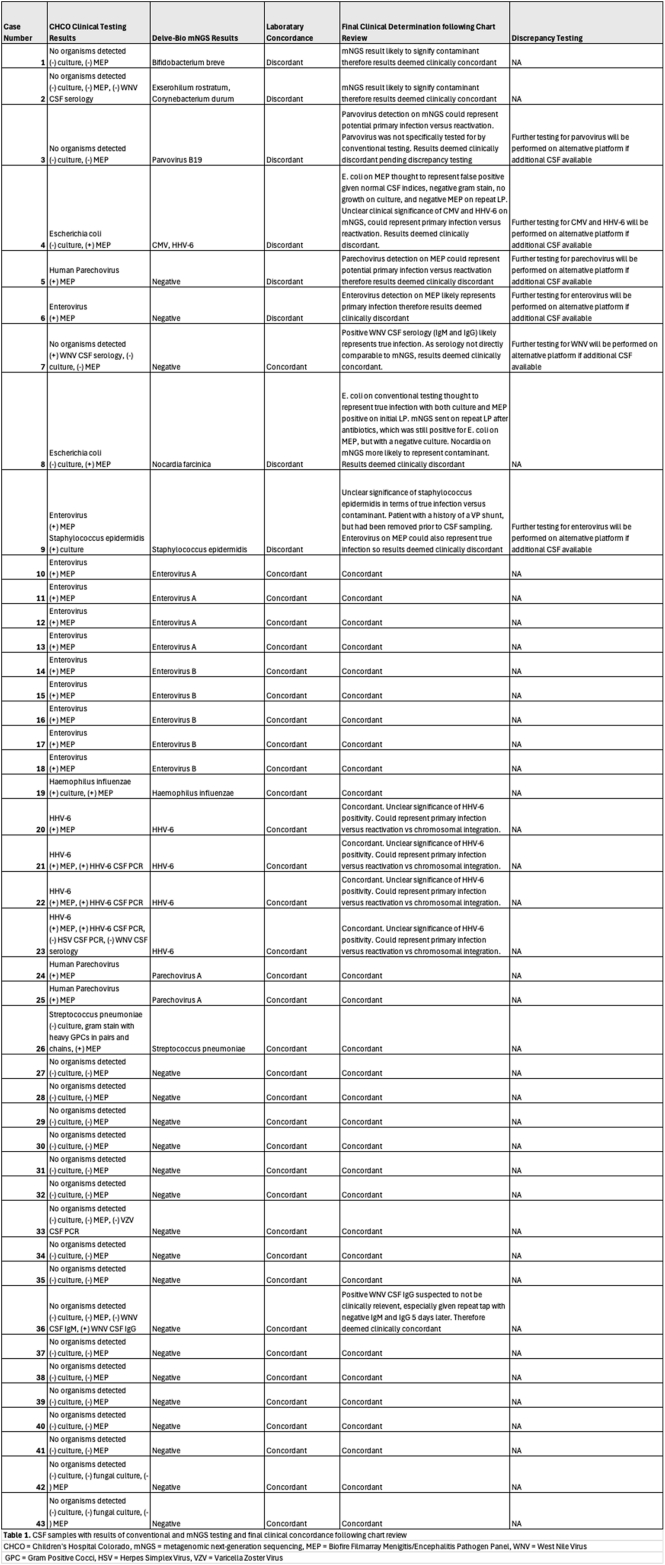

**Figure:**
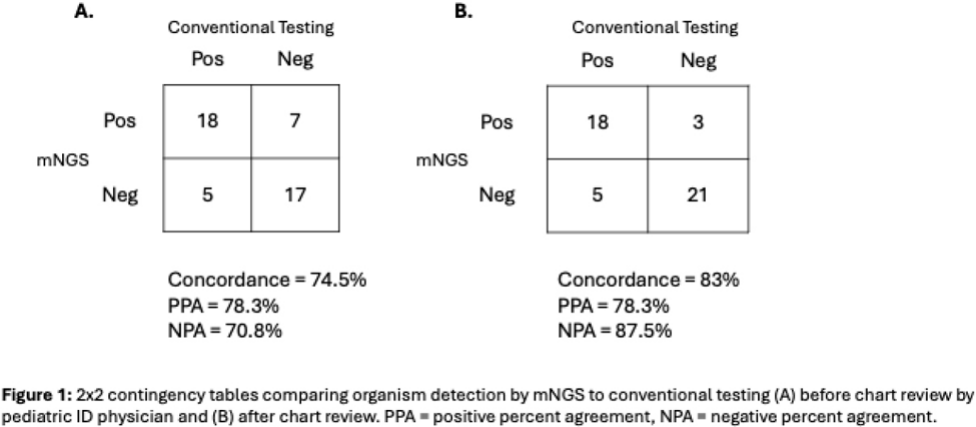

**Methods:**

Frozen residual CSF samples from children hospitalized at CHCO with suspected CNS infection were tested on the Delve Detect mNGS platform. Laboratory concordance, including positive and negative percent agreement (PPA; NPA), was calculated between mNGS and clinically conducted conventional microbiologic testing results (culture, single- and multi-plex PCR, antigen) with blinded infectious diseases physician chart review to adjudicate clinical diagnosis for clinical concordance. A third independent assay was used for discrepancy testing on residual samples when feasible.

**Results:**

mNGS identified 25 organisms in 23 of 43 (53%) samples, compared to 23 organisms in 22 (51%) samples with conventional methods. Laboratory concordance between testing methods was 74.5%. PPA and NPA were 78.3% and 70.8% respectively. There were 5 organisms (10.6%) identified by conventional methods that were not detected by mNGS and 7 organisms (14.9%) detected by mNGS that were not identified by conventional methods.

Clinical concordance following chart review was 83%. 4 non-pathogenic organisms identified by mNGS were considered clinically concordant with negative conventional testing. Subsequent PPA and NPA were 78.3% and 87.5%. Results of discrepancy testing are pending.

**Conclusion:**

This study supports the analytical validity and potential utility of using mNGS as an adjunct to conventional microbiological testing for the diagnosis of suspected CNS infections in children. Diagnostic stewardship, including careful clinical interpretation of mNGS results, is essential due to the high sensitivity and unbiased nature of mNGS testing.

**Disclosures:**

Samuel Showalter, MD, Delve Bio: Grant/Research Support Timothy M. Blicharz, PhD, Delve Bio, Inc.: Employee|Delve Bio, Inc.: Stocks/Bonds (Private Company) Steve Miller, MD/PhD, Delve Bio: Employee|Delve Bio: Stocks/Bonds (Private Company) Sudeb Dalai, MD PhD, Delve Bio: Stocks/Bonds (Private Company) Samuel R. Dominguez, MD, PhD, Biofire Diagnostics: Advisor/Consultant|Biofire Diagnostics: Grant/Research Support|DelveBio: Advisor/Consultant|DelveBio: Grant/Research Support|Karius: Advisor/Consultant|Karius: Grant/Research Support|Roche: Advisor/Consultant

